# CK2α Deletion in the Hematopoietic Compartment Shows a Mild Alteration in Terminally Differentiated Cells and the Expansion of Stem Cells

**DOI:** 10.3390/cells14130963

**Published:** 2025-06-24

**Authors:** Rajesh Rajaiah, Muhammad Daniyal, Marudhu Pandiyan Shanmugam, Hannah Valensi, Koby Duke, Katherine Mercer, Morgann Klink, Matthew Lanza, Yasin Uzun, Suming Huang, Sinisa Dovat, Chandrika Gowda Behura

**Affiliations:** 1 Department of Pediatrics, Milton S. Hershey Medical Center, Pennsylvania State University College of Medicine Hershey, Hershey, PA 17033, USA; rqr5616@psu.edu (R.R.); mdanial@pennstatehealth.psu.edu (M.D.); mshanmugam@pennstatehealth.psu.edu (M.P.S.); hvalensi@pennstatehealth.psu.edu (H.V.); kduke@pennstatehealth.psu.edu (K.D.); kmercer1@pennstatehealth.psu.edu (K.M.); mreed7@pennstatehealth.psu.edu (M.K.); yuzun@pennstatehealth.psu.edu (Y.U.); shuang4@pennstatehealth.psu.edu (S.H.); sdovat@pennstatehealth.psu.edu (S.D.); 2 Department of Comparative Medicine, Pennsylvania State University College of Medicine Hershey, Hershey, PA 17033, USA; mlanza1@pennstatehealth.psu.edu

**Keywords:** casein kinase 2, normal hematopoiesis, hematopoietic stem cells, Vav iCre, conditional knockout, CK2alpha, HSC, mouse hematopoiesis

## Abstract

Casein Kinase II (CK2) is a ubiquitously present serine/threonine kinase essential for mammalian development. CK2 holoenzyme is a tetramer with two highly related catalytic subunits (α or α’) and two regulatory ß subunits. Global deletion of the α or β subunit in mice is embryonically lethal. We and others have shown that CK2 is overexpressed in leukemia cells and plays an important role in cell cycle, survival, and resistance to the apoptosis of leukemia stem cells (LSCs). To study the role of CK2α in adult mouse hematopoiesis, we generated hematopoietic cell-specific CK2α-conditional knockout mice (Vav-iCreCK2 ^f/f^). Here we report the generation and validation of a novel mouse model that lacks CK2α in the hematopoietic compartment. Vav-iCreCK2α ^f/f^ mice were viable without dysmorphic features and showed a mild phenotype under baseline conditions. In Vav-iCreCK2α ^f/f^ mice, the blood count showed a significant decrease in total red blood cells and platelets. The spleen was enlarged in Vav-iCreCK2α ^f/f^ mice with evidence of extramedullary hematopoiesis. HSC and early progenitor cell compartments showed expansion in CK2α-null bone marrow, suggesting that the absence of CK2α impaired their proliferation and differentiation. Given the established roles of CK2 in cell cycle regulation and the findings reported here, further functional studies are warranted to investigate the role of CK2α in HSC self-renewal and differentiation. This mouse model serves as a valuable tool for understanding the role of CK2α in normal and malignant hematopoiesis.

## 1. Introduction

CK2 is a ubiquitous and pleotropic serine/threonine kinase known to phosphorylate nearly one fifth of the human kinome [1,2,3,4]. Although the CK2 holoenzyme exists as a tetramer composed of two catalytic subunits (CK2α and/or CK2α′) and two regulatory (CK2β) subunits, each subunit also shows independent and distinct function [5]. CK2α and CK2α′ are encoded by *CSNK2A1* and *CSNK2A2* genes, respectively, sharing 90% similarity within their catalytic domains [6]. The CK2β regulatory subunit is encoded by the *CSNK2B* gene and plays a role in maintaining the stability of the holoenzyme, substrate specificity [2,4,7,8]. CK2 is essential for embryonic development and the global deletion of either CK2α or CK2β in mice is embryonic lethal [9,10]. CK2α’ deletion causes azoospermia in mice but no other abnormalities, suggesting that CK2α compensates for its loss. It is well established that CK2 is involved in a variety of physiological cellular processes and signaling pathways, including transcription, translation, cell cycle progression, protein stability, and degradation [3,11,12,13]. In addition, CK2 is implicated in the pathogenesis of several human diseases, including a variety of cancers, inflammatory diseases, autoimmune and infectious diseases [3,14,15]. CK2 exerts its action by enhancing cell survival and proliferation, tumor progression and angiogenesis [1,16,17,18]. Specifically, in tumor cells, CK2 sustains pro-survival and proliferative signaling cascades that depend on NF-κB, PI3K/AKT/mTOR and/or JAK/STAT pathways [3,15,19]. As a result, CK2 is extensively studied as a therapeutic target in human diseases, including cancer. 

Normal hematopoiesis is tightly regulated by complex intrinsic and extrinsic mechanisms [20,21]. Transcription factors such as IKAROS, PTEN, PU.1, and PML that regulate various aspects of normal and malignant hematopoiesis are phosphorylated by CK2 [22,23,24,25,26,27]. The CK2-mediated phosphorylation of Ikaros, a master regulator of lymphoid hematopoiesis, impairs its stability, localization, and DNA binding [28,29]. CK2 promotes leukemogenesis in B-cell acute lymphoblastic leukemia mainly by the functional inactivation of the Ikaros tumor suppressor [30]. We and others have shown that, in hematopoietic malignancies, CK2 promotes leukemia stem cell (LSC) survival, resistance to apoptosis, and chemosensitivity [15,31,32,33,34,35,36,37]. The pharmacological inhibitor of CK2, CX-4945 (silmitasertib), selectively kills leukemia cells with minimal cytotoxic effects on normal bone marrow cells [15,32,38,39]. However, the physiologic role of CK2α in adult mouse hematopoiesis, specifically HSC and precursor stem cell development, is not well studied. A recent study from Tubi et al. showed that CK2β is essential for HSC differentiation and erythropoiesis [40]. Myeloid cell development was not significantly affected by CK2α deficiency in the conditional knockout model [41]. The deletion of CK2α in mature T cells notably impaired Th17 cell polarization and led to increased regulatory T cells [18,42]. It is known that the CK2 subunits (ck2α, ck2α’, ck2β) show kinase activity independently from the tetrameric holoenzyme and have distinct functions [16]. In order to study the effect of CK2α deletion on HSC, HPSC, and differentiated cells, we generated hematopoietic compartment-specific CK2α-conditional knockout (Vav-iCre CK2α^f/f^) mice by crossing csnk2a1^f/f^ loxP mice with the Cre recombinase under the control of the Vav1 promoter [43]. We hypothesized that, unlike CK2β, CK2α is dispensable for normal hematopoiesis, but CK2α deficiency may reveal alterations in HSC and HPSC phenotypes as well as molecular signature. A better understanding of the physiological role of CK2α in normal hematopoiesis is critical for further investigating the role of CK2α in malignant hematopoiesis. A CK2 inhibitor is in clinical testing for several cancers. Accumulating preclinical data about the therapeutic efficacy of CK2 inhibitors in hematological malignancies is encouraging [35,38]. The genetically engineered mouse model (GEMM) of CK2α KO described here, along with other GEMMs of leukemia [44,45,46], will accelerate the creation of studies to establish the pro-oncogenic role of CK2α in leukemia development. 

## 2. Materials and Methods

### 2.1. Animals

Mice with floxed *csnk2a1* alleles were a kind gift from Drs. Flajolet and Rebholz (City College of New York, New York, NY, USA) [47]. Vav-iCre mice that express improved Cre recombinase (iCre) under the control of the mouse vav 1 (Vav1) promoter were purchased from The Jackson Laboratory [48]. The seven wild type (WT) Vav-CreCK2α^+/+^, eight knockout (KO) Vav-iCreCK2α^f/f^ and four heterozygous (Het) Vav-iCreCK2α^f/+^ mice used in this study were 8-to-12-week-old littermates, both male and female. These mice were crossed on a C57BL/6 background for at least three generations. Littermates of the Vav-iCreCK2α^f/f^ (KO) were used as wild type controls in each specific experiment. The deletion of csnk2a1 was confirmed by genotyping performed using real-time PCR (TransnetYX, Inc. Cordova, TN, USA). Primers are listed in Table S1. All of the mice were bred and maintained in pathogen-free conditions at the Penn State College of Medicine’s comparative medicine facility. The protocols were approved by the Institutional Animal Care and Use Committee of Penn State University.

### 2.2. Quantitative Real-Time PCR

Total RNA was isolated from *Vav-iCreCK2α^+/+^, Vav-iCreCK2α^f/f^* and *Vav-iCreCK2α^f/+^* mice bone marrow (BM) and spleen total mononuclear cells without RBC lysis using TRIzol reagent (Invitrogen, Carlsbad, CA, USA) and reverse transcribed to cDNA using the iScript cDNA synthesis kit (BioRad, Hercules, CA, USA). Mouse specific csnk2a1 expression was quantitated in QuantStudio 3 (Applied Biosystems, Carlsbad, CA, USA) using the Taqman primers/probe set, CK2α (Integrated DNA Technologies, Coralville, IA, USA). Mouse GAPDH (Integrated DNA Technologies, Coralville, IA, USA) served as a house keeping gene. 

### 2.3. Western Blotting Analysis

A whole-cell lysate was prepared from BM and spleen cells from the Vav-iCreCK2α^+/+^, Vav-iCreCK2α^f/f^ and Vav-iCreCK2α^f/+^ mice without RBC lysis using a cell lysis buffer (Cell Signaling Technology, Boston, MA, USA). Initially, 20 μg of protein from each sample was subjected to Western blot analysis with the β-actin antibody and band intensities were normalized. Protein from normalized cell lysates was separated by 4–20% SDS-PAGE in Laemmli buffer and electro-transferred to a PVDF membrane. Further, the membranes were blocked with TBST (20 mmol/L Tris, pH 7.5 with 150 mmol/L NaCl and 0.05% Tween-20) containing 5% non-fat milk powder for 1h. Then, the blot was probed with primary antibodies specific for mouse CK2α and CK2α’ (Santa Cruz, Dallas, TX, USA) overnight at 4 °C. Membranes were washed extensively (4×) using TBST and incubated with HRP-conjugated secondary antibodies (Cell Signaling Technology, Boston, MA, USA) for one hour at room temperature. The blots were developed with an enhanced chemiluminescence substrate and visualized using a Bioimaging system (Bio-Rad, Hercules, CA, USA). Mouse anti-CK2α, -CK2β, and -CK2α’ antibodies were used at a 1:10,000 dilution and a secondary antibody was used at dilution of 1:3000. In order to discriminate between overall CK2 kinase activity and CK2α-specific activity, we evaluated phosphorylation of known natural substrates of CK2α such as Akt serine 129 and NFκBp65. The antibodies used are listed in Supplemental (Table S2). 

### 2.4. Flow Cytometry

The BM and spleen cells from the Vav-iCreCK2α^+/+^, Vav-iCreCK2α^f/f^ and Vav-iCreCK2α^f/+^ mice were isolated, passed through a 40 µm nylon cell strainer (Falcon, Mesa, AZ, USA), treated with RBC lysis solution to remove RBCs, and resuspended in PBS supplemented with 3% FBS. Aqua zombie was used for gating live dead cells purchased from Bio Legend, CA, USA. For the analysis of the erythroid panel, BV711 anti-CD45, FITC anti-Ter119, and PE anti-CD71 anti-mouse Abs were purchased from Bio legend, San Diego, CA, USA. For the HSC panel, BV 421 anti-CD135, FITC anti-Lin1, APC anti-c-Kit, APC-Cy7 anti-CD127/IL-7Ra, PE-Cy5 anti-CD34, PE anti-Sca1, and PE-Cy7 anti-CD16/32 anti-mouse Abs were purchased from Bio Legends, San Diego, CA, USA. The immunophenotyping panel flow antibodies, PE-Cy5 anti-CD45, APC anti-CD3, BV421 anti-CD19, PE-Texas Red anti-NKp46, BV605 anti-CD11b, BV711 anti-F4/80, APC-Cy7 anti-CD11c, Alexa Flour anti-MHC-II, PE-Cy7 anti-Ly6G, and FITC anti-Ly6C, were purchased from Bio Legends, San Diego, CA, USA. Flow cytometric analysis was performed using BD Fortessa 16 (BD Biosciences, San Jose, CA, USA) and fluorescence-activated cell sorting (FACS) data were analyzed using FlowJo software v10 (Tree Star, Ashland, OR). 

### 2.5. Quantitation of Cell Frequency and Absolute Number

The frequencies of different cell types in the bone marrow and spleen samples were calculated by FACS analysis. Absolute cell numbers were calculated based on the total cell numbers using a hemocytometer. The absolute number of cells in the populations of interest was calculated using the formula absolute cell number = (number of cells in gate/number of cells in live cells) × total cell number in organs (BM or spleen).

### 2.6. Histology

After removal of the muscle tissues, the femurs were fixed and decalcified in fixative decalcifier (StatLab, Hershey, PA, USA) at room temperature for 48 h. Simultaneously, spleen tissues were fixed in 4% formaldehyde for 48 h. The tissues were processed and embedded in paraffin. The tissues were sectioned at 5 µm and stained with hematoxylin and eosin (H&E). Slides were analyzed for tissue architecture and hematopoiesis (M:E ratio) by pathologists. 

### 2.7. Bulk RNA Sequencing 

Total RNA was isolated from the BM and spleen cells from the Vav-CreCK2α^+/+^, Vav-CreCK2α^f/f^, and Vav-CreCK2α^f/+^ mice using TRIzol reagent (Invitrogen, Carlsbad, CA, USA) and quantitated using a NanoDrop One Microvolume UV-Vis Spectrophotometer (Thermo Fisher, Waltham, MA, USA). Paired-end RNA sequencing reads were trimmed using Trimmomatic [49] with the TruSeq3_PE fa adapter sequences to remove adapters and low-quality bases. The trimmed reads were then aligned to the corresponding reference genome (mm39) using the STAR aligner [50]. Gene-level quantification was performed based on the Gencode annotation [51], using version 36. Gene expression levels were normalized and expressed as fragments per kilobase of transcript per million mapped reads (FPKM) using Cufflinks [52]. For downstream analyses, genes with an FPKM value greater than 1.0 in at least two samples within either experimental group were retained.

Raw read counts were obtained using the Counts function from the Rsubread package [53]. Differential expression analysis was conducted using edgeR [54], where a quasi-likelihood negative binomial generalized log-linear model was fitted to the data via the glmQLFTest function. Prior to modeling, raw count data were normalized using the trimmed mean of M-values (TMMs) method. The Benjamini–Hochberg (BH) procedure was applied for multiple testing correction, and genes with an adjusted *p*-value below 0.05 were considered significantly differentially expressed.

Gene set enrichment analysis (GSEA) was performed using GSEA software v4.4.0 [55], with normalized fragments per kilobase of transcript per million mapped reads (FPKM) values as the input. Functional enrichment analysis via Metascape [56] was conducted using the top 100 upregulated and top 100 downregulated genes, ranked by statistical significance. A visualization of differential expression and pathway enrichment results was performed using the ggplot2, pheatmap, enrichplot, and dplyr packages in R.

## 3. Results

### 3.1. Loss of CK2α in the Hematopoietic Compartment Shows a Mild Phenotype in Adult Mice

**Figure 1:**
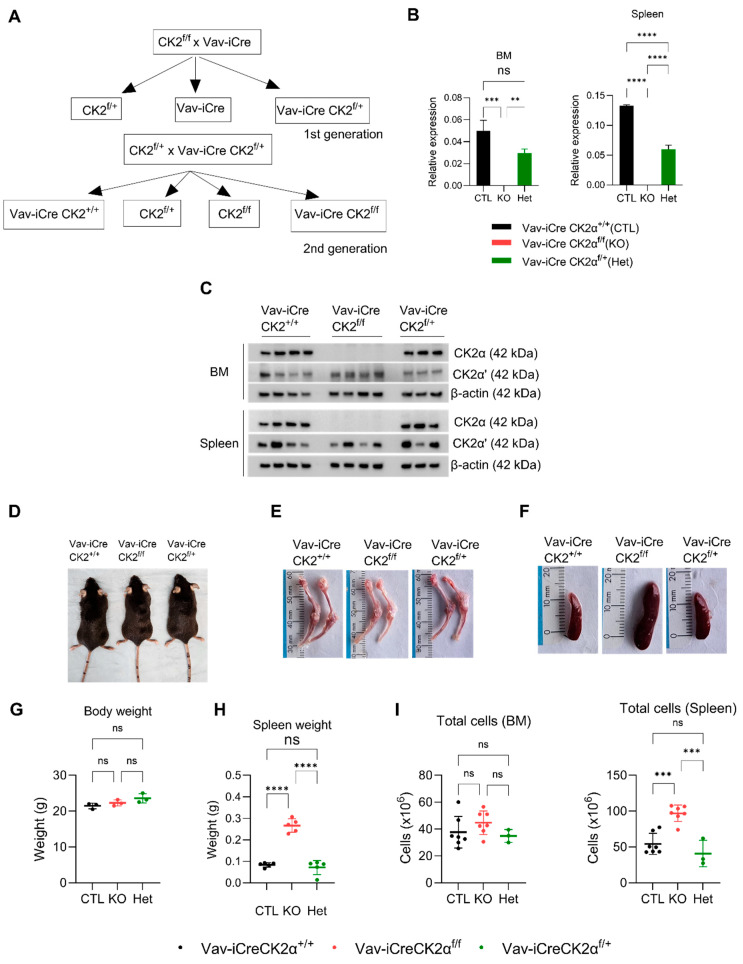
CK2α knockout mice generation and morphologic evaluation. (**A**) Schema of mouse breeding to generate Vav-iCre-CK2α^f/f^. (**B**,**C**) mRNA and protein expression of CK2α in four WT (Vav-iCreCK2α^+/+^), four cKO (Vav-iCreCK2^f/f^), and three Het (Vav-iCreCk2a^f/+^) mice bone marrow and spleen samples. (**D**), A representative photograph of WT, CK2α KO, and Het mice. (**E**,**F**) Photographs of bone (femur and tibia) and spleen from WT, CK2α KO, and Het mice. (**G**) Body weight in grams of adult WT, CK2α KO, and Het mice. (**H**) Spleen weight in grams of adult WT, CK2α KO and Het mice. (**I**), Total number of mononuclear cells in BM and spleen cells in WT, KO and Het mice. ns: non-significance, **: *p* < 0.01, ***: *p* < 0.001, ****: *p* < 0.0001.

Since the germline deletion of CK2α in mice is embryonically lethal, we generated a hematopoietic tissue-specific CK2α knockout mouse. We generated Vav1-Cre CK2α^f/f^ by crossing CK2α^f/f^ mice with Vav-iCre as shown in the schema (Figure 1A). The wild type (WT) *Vav-iCreCK2α^+/+^*, knockout (cKO) *Vav-iCreCK2α^f/f,^* and heterozygous (Het) Vav-iCreCK2α^f/+^ pups were born after normal gestation, and no intrauterine fetal loss was noted. CK2α cKO pups were viable at birth and showed no difference in appearance or weight compared to their littermates. The genotype of the mice was confirmed using real-time PCR. The absence of CK2αmRNA expression and protein expression in the target tissues (BM and spleen) of the CK2αcKO mice was confirmed by qRT-PCR and Western blotting (Figure 1B,C). While CK2α protein expression was absent in the CK2α cKO mice, there was no significant change in CK2α’ expression in the bone marrow and variable expression in the spleen tissue (Figure 1C). More importantly, there was no compensatory increase in CK2α’ expression. The CK2β regulatory subunit protein level was mildly decreased in both bone marrow and spleen cells from CK2α cKO mice, which is consistent with previous reports (Figure 1C) [57]. A significant decrease in CK2α activity measured using CK2α-specific substrate (Akt serine 129, NFκBp65 serine 529) phosphorylation was also confirmed in target tissues from the WT, CK2α cKO, and Het mice (Figure S1A). CK2α cKO offspring showed a normal Mendelian distribution, normal development, normal morphology, body weight, and normal life span compared to their control littermates (Vav-iCre CK2α^+/+^) (Figure 1D). Both male and female adult mice aged 8–12 weeks were used for analysis. CK2α cKO mice had pale looking bones compared to the control littermates’ bones (Figure 1E). CK2α cKO mice demonstrate an enlarged spleen and increased spleen weight without any notable change in their whole-body weight compared to the control littermates (Figure 1F–H). The total mononuclear cells in the spleen samples was increased in the CK2α cKO mice (Figure 1I). However, there was slight increase (not statistically significant) in total mononuclear cells in the bone marrow of the CK2α-deficient mice compared to the control mice. In summary, CK2α knockout in the hematopoietic compartment leads to mild hematopoietic phenotype changes.

### 3.2. CK2α-Deficient Mice Show Splenomegaly, and Extramedullary Erythropoiesis

**Figure 2:**
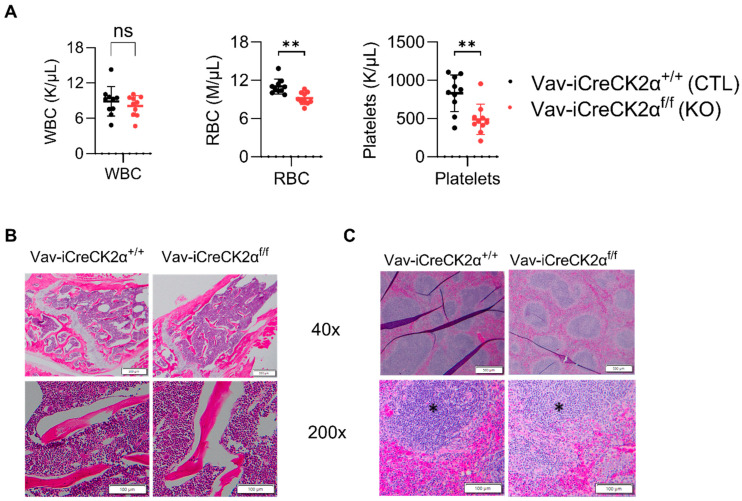
Complete blood count and tissue histology changes in CK2α cKO mice. (**A**) Blood from WT and KO mice was collected in tubes with K2E, and complete blood count was performed in a Hemavet hematological analyzer. Bone (**B**) and spleen (**C**) cells stained with hematoxylin and eosin, and Images were captured at 40× and 200× under a light microscope. Scale bars: 50–100 μm. * Indicates white pulp. ns: non-significance, **: *p* < 0.01.

Complete blood counts from peripheral blood showed a decrease in the total red blood cell count and platelet counts in the CK2α cKO mice compared to the control mice (Figure 2A). These results suggest that CK2α has a non-essential functional role in mouse erythropoiesis. H&E staining of the CK2α WT mice BM exhibited normal cellularity with the presence of all cell lines and normal maturation, and a normal myeloid to erythroid ratio (M:E) (1.5:1 to 2:1) (Figure 2B). However, the CK2α cKO mouse BM demonstrated severe myeloid hyperplasia, with an abundance of myeloid lineage cells and mature granulocytes (Figure 2B). Moreover, the M:E ratio was greatly increased (greater than 10:1) in CK2α cKO mice BM (Figure 2B). The population of erythroid and lymphoid cell lines decreased, but true erythroid hypoplasia was not evident by histology. Spleen from both CK2α WT and CK2α cKO mice demonstrated extramedullary hematopoiesis within the red pulp. Although this is a normal finding in mice, the amount of extramedullary hematopoiesis was increased within the CK2α cKO spleen sample. The CK2α cKO mouse spleen was also characterized by relatively increased myelopoiesis (Figure 2C). Furthermore, the CK2α cKO mouse spleen had fewer mature lymphocytes within the white pulp. Overall, the findings show that the CK2α spleen is enlarged due to extramedullary hematopoiesis and increased myelopoiesis relative to erythropoiesis. Interestingly, the megakaryocyte and peripheral platelet cell counts are significantly decreased.

### 3.3. CK2α cKO Mice Show Mildly Impaired Erythropoiesis

**Figure 3:**
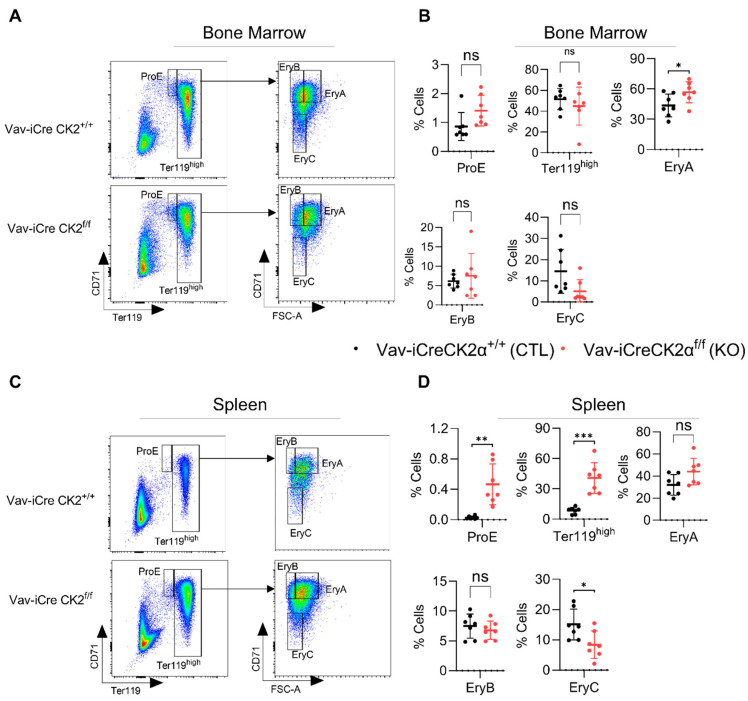
Analyses of erythroid compartment in bone marrow and spleens of CK2α KO and WT mice. Flow cytometry dot-plots showing gating strategies for ProE, Ter119^high^, EryA, EryB and EryC from WT and CK2α KO mice BM (**A**) and spleens (**C**). Percentage of ProE, Ter119^high^, EryA, EryB and EryC cells from WT, CK2α KO BM (**B**) and spleens (**D**) were analyzed by FACS. ns: non-significance, *: *p* ≤ 0.05, **: *p* < 0.01, ***: *p* < 0.001.

CK2α KO mice exhibited relatively pale bones compared to the WT mice (Figure 1E), suggesting a paucity of erythroid cells. Proerythroblasts (ProE, CD71^+^Ter119^lo^), erythroblast A (EryA, CD71^hi^Ter119^hi^FSC^hi^), EryB (CD71^hi^Ter119^hi^FSC^lo^), and EryC (CD71^lo^Ter119^hi^FSC^lo^) cells were gated based on the expression of CD71 and Ter119 (Figure 3A,C). Ter119 is a marker of late-stage mouse erythroid cells and mature erythrocytes. The complete blood count revealed significantly decreased the total red blood cell count (Figure 2A) and bone histology noted an increased M:E ratio and mild erythroid hypoplasia (Figure 2B). FACS analyses revealed alterations in subsets of erythroid precursor cells. CK2α cKO bone marrow showed increased (statistically nonsignificant) ProE and unaltered numbers of Ter119^hi^ erythroblasts (Figure 3B). However, there was an increased number of subsequent erythroblast (Ery) A cells and decreased Ery C cells in the BM (Figure 3B). EryA, B and C originated from early progenitor ProE via distinct stages in the development of the erythroid panel. EryA represents early basophilic erythroblasts, EryB represents late basophilic polychromatic and orthochromatic erythroblasts and EryC represents orthochromatic erythroblasts with mature erythrocytes. A significantly increased number of ProE, Ter119^hi^ and decreased frequency of EryC cells was observed in the spleen samples from CK2α cKO mice (Figure 3D). Extramedullary hematopoiesis in the spleen is a common phenomenon of activation of erythropoiesis during a stress conditions [58]. Moreover, the increased spleen size in CK2α cKO mice (Figure 1F) likely suggests compensatory extramedullary erythropoiesis (Figure 3C,D).

### 3.4. CK2α cKO Mice Show Expansion of HSC and Progenitor Cells

**Figure 4:**
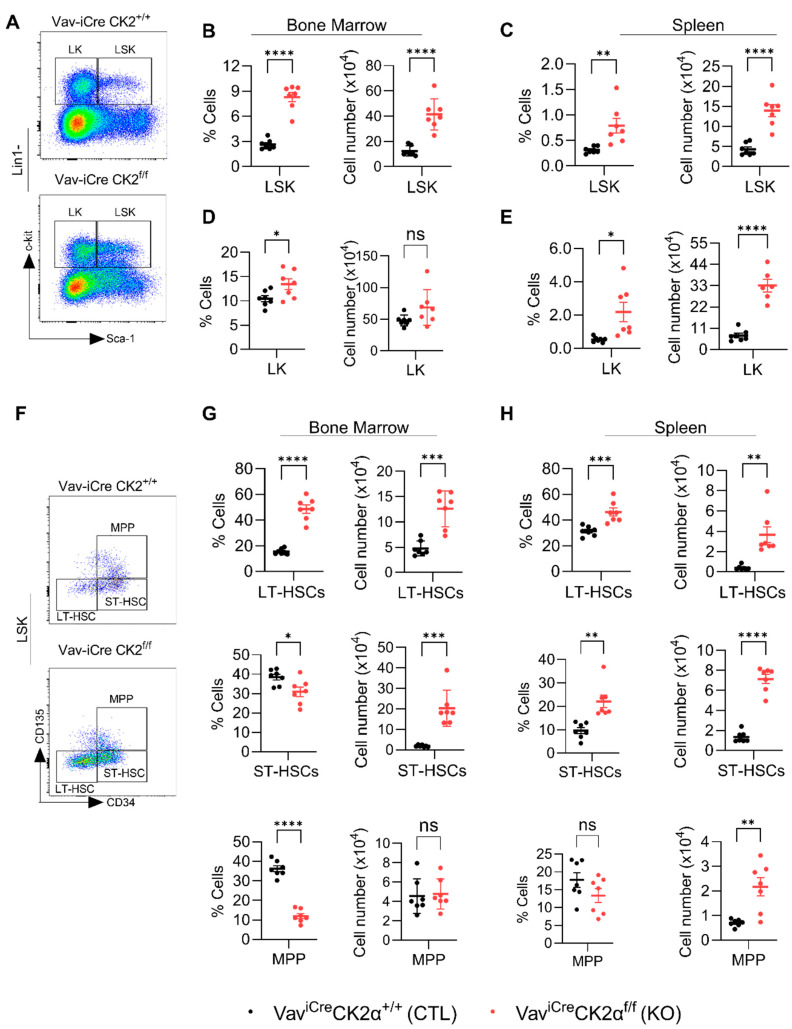
Analyses of hematopoietic stem cells and early progenitor cells. BM and spleen cells were isolated and surface-stained with different fluorochrome-labeled antibodies and analyzed for different hematopoietic lineages. (**A**) A representative gating strategy for LSK (Lin1^−^Sca1^+^c-kit^+^) and LK (Lin1^−^Sca1^−^c-kit^+^) cells from WT and CK2α KO mice BM. (**B**,**C**), Percentage and absolute number of LSK cells from WT and CK2α KO mouse BM (**B**) and spleen (**C**) samples. (**D**,**E**), Percentage and absolute number of LK cells from WT and CK2α KO mice BM (**D**) and spleen (**E**) and spleen. (**F**), A representative gating strategy for MPP, ST-HSCs and LT-HSCs (from LSK cells) from WT and CK2α KO mouse BM. (**G**,**H**), Percentage and absolute number of MPP, ST-HSCs and LT-HSCs from WT and CK2α KO mouse BM (**G**) and spleen samples (**H**). ns: non-significance, *: *p* ≤ 0.05, **: *p* < 0.01, ***: *p* < 0.001, ****: *p* < 0.0001.

To check the effect of CK2α deficiency on pluripotent stem cells and progenitors, we performed FACS analyses of HSPCs using an established gating strategy (Figure S2 and Figure 4A) [59]. The relative proportion and absolute number of the LSK (Lin^−^Sca1^+^c-Kit1^+^) population in the BM (Figure 4B) and spleens (Figure 4C) of CK2α KO mice were significantly increased. The relative proportion of LK (Lin^−^Sca1^−^c-Kit1^+^) cells was high in CK2α KO BM, but the absolute number was unchanged (Figure 4D). On the other hand, both the relative proportion and absolute number of LK cells were significantly high in CK2α KO mice spleen compared to the WT mice (Figure 4E). LSK cells mainly demonstrated their ability to generate blood cells and reconstitute the hematopoietic system when it was damaged. On the other hand, LK cells are committed erythromyeloid progenitors that are responsible for replenishing common myeloid progenitors (CMPs), granulocyte–monocyte progenitors (GMPs), and megakaryocyte–erythrocyte progenitors (MEPs).

The LSK population is heterogeneous, with a hematopoietic population that includes long-term HSCs (LT-HSCs), short-term HSCs (ST-HSCs), and multipotent progenitors (MPPs) (Figure 4F). Within the LSK population, the relative proportion and absolute number of LT-HSC increased in both BM and spleen of CK2α KO mice (Figure 4G,H). On the other hand, the relative proportion of ST-HSCs in CK2α KO mouse BM was decreased (Figure 4G), but the absolute number was comparably higher than that in the control mouse BM (Figure 4G). However, both the relative proportion and absolute number of ST-HSCs in the CK2α KO mouse spleens were significantly increased (Figure 4H). There was a reduction in the relative proportion of MPPs (Figure 4G). Although the relative proportion of spleen MPPs was not altered in the CK2α KO mice, the absolute number was increased compared to the WT mouse spleen samples (Figure 4H). Under normal physiological conditions, LT-HSCs exist in a resting state and become activated when the body is stimulated by stress [60]. Moreover, an increased number of LT-HSCs has been reported in leukemic conditions. The increased number of LT-HSCs in the CK2α KO mice may indicate that CK2α plays an essential role in maintaining normal physiology and may be required for the maintenance of LT-HSCs in quiescence. 

**Figure 5:**
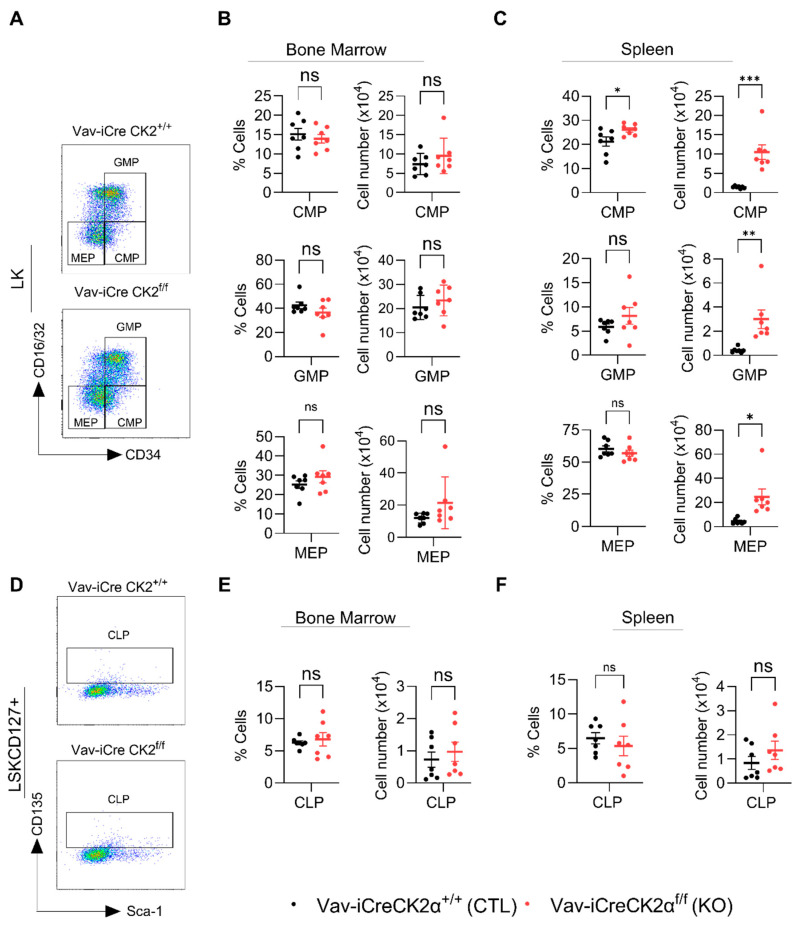
Analyses of lineage-specific progenitor cells. A representative gating strategy for CMP, GMP and MEP cells from WT and CK2α KO mice (**A**). Percentage and absolute number of CMP, GMP and MEP (from LK cells) cells from WT and CK2α KO mouse BM (**B**) and spleen (**C**) samples. A representative gating strategy for CLP (LSKCD127+ cells) from WT and CK2α KO mice (**D**). Percentage and absolute number of CLP from WT and CK2α KO mouse BM (**E**) and spleen (**F**) samples. ns: non-significance, *: *p* ≤ 0.05, **: *p* < 0.01, ***: *p* < 0.001.

LK cells are lineage-committed erythromyeloid progenitors that are composed of common myeloid progenitors (CMPs), granulocyte–monocyte progenitors (GMPs), and megakaryocyte–erythrocyte progenitors (MEPs) (Figure 5A). FACS analyses of the LK subpopulations revealed that both the relative proportion and absolute numbers of CMPs, GMPs, and MEPs in CK2α KO BM were not altered compared to the WT mouse BM (Figure 5B). On the other hand, the relative proportions of CMPs, GMPs, and MEPs in CK2α KO spleens were comparable with those in the WT mice (Figure 5C). Both the relative and absolute numbers of CMPs were significantly elevated in the CK2α KO mouse spleens compared to the WT mouse spleens (Figure 5C). While the relative proportion of GMPs and MEPs was not altered in the CK2α KO mouse spleens, the absolute number was significantly higher compared to that in the WT mice (Figure 5C). Further, the relative proportion and absolute numbers of common lymphoid progenitors (CLPs; LSKCD127^+^) (Figure 5D) which are different in lineage from CMPs, GMPs, and MEPs were not altered in the CK2α KO mouse BM or spleen samples (Figure 5E,F). These findings indicate that a loss of *CK2α* differentially affects HSPCs’ hematopoietic differentiation ability. Moreover, a loss of CK2α causes the expansion of HSCs in the BM and spleen and an increase in myeloid progenitors in the spleen.

### 3.5. Loss of CK2α Differentially Regulate Immune Cells and Enhances Myelopoiesis in Mice

**Figure 6:**
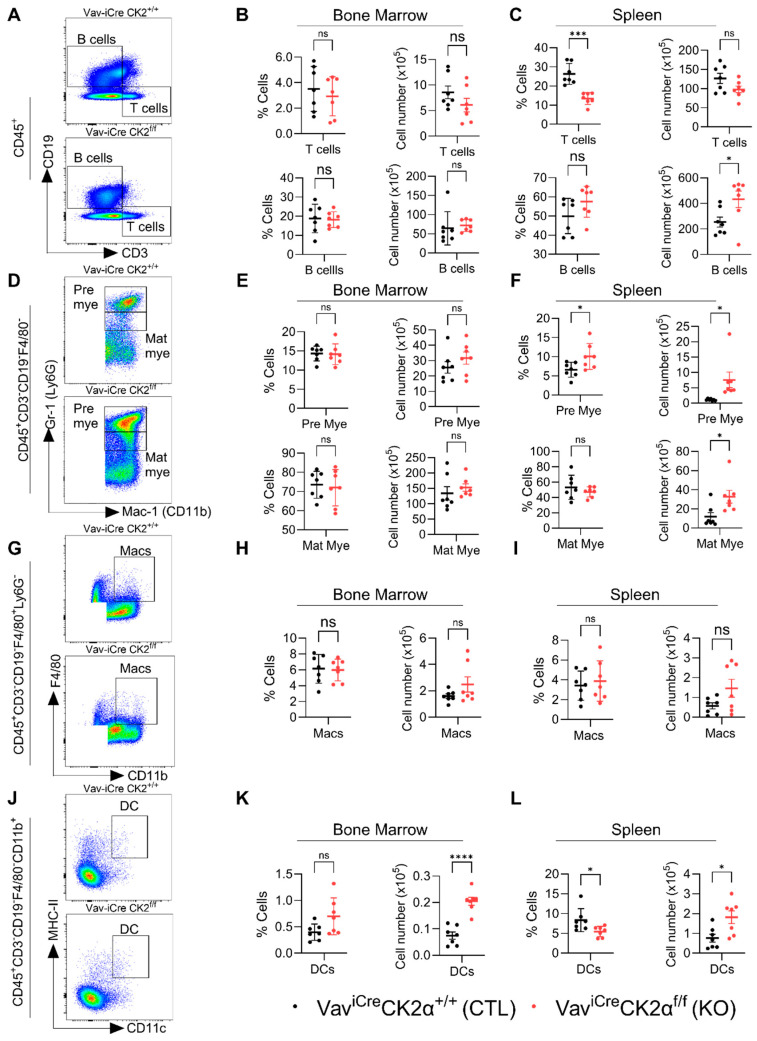
Analyses of immune cell compartments. (**A**) A representative gating strategy for T and B cells from WT and CK2α KO mice. (**B**,**C**) Percentage and absolute number of T and B cells from WT and CK2α KO mouse BM (**B**) and spleen (**C**) samples. (**D**) An illustrative gating strategy for pre and matured myeloid cells (from CD45 + CD3-CD19-F480- population) from WT and CK2α KO mice. (**E**,**F**) Percentage and absolute number of pre-mature myeloid (pre mye) and mature myeloid (mat mye) cells from WT and CK2α KO mouse BM (**E**) and spleen (**F**) samples. (**G**) A representative gating strategy for macrophages (from CD45 + CD3-CD19-F4/80+ Ly6G- population) from WT and CK2α KO mice. (**H**,**I**) Percentage and absolute number of macrophages (Macs) from WT and CK2α KO mouse BM (**H**) and spleen (**I**) samples. (**J**) An illustrative gating strategy for dendritic cells (from CD45 + CD3-CD19-F4/80-CD11b+ population) from WT and CK2α KO mice. (**K**,**L**) Percentage and absolute number of dendritic cells (DCs) from WT and CK2α KO mouse BM (**K**) and spleen (**L**) samples. ns: non-significance, *: *p* ≤ 0.05, ***: *p* < 0.001, ****: *p* < 0.0001.

Next, we analyzed the changes in the composition of terminally differentiated immune cells in CK2 cKO mice compared to the controls (Figure 6A,D,G,J). CK2α deficiency did not alter the relative proportion and absolute number of T and B cells in CK2α KO mouse BM (Figure 6B), whereas the relative proportion of T cells in the CK2α KO spleen samples was significantly decreased without altering the absolute numbers in the CK2α KO spleen samples compared to the WT mice (Figure 6C). An increased relative proportion of B cells was observed in the CK2α KO mouse spleen samples, but it was not significant, unlike the absolute numbers, which were significantly elevated compared to the control mice (Figure 6C). Further, the levels of pre- and matured myeloid cells were not altered in the CK2α KO mouse BM (Figure 6E). However, both pre- and matured myeloid cells populations were increased in CK2α KO mice spleen compared to WT mice (Figure 6F). While the macrophage cell population levels were unchanged in the CK2α KO mouse BM and spleen samples (Figure 6H,I), the dendritic cell (DC) population was increased in both the BM and spleens of the CK2α KO mice compared to the WT mice (Figure 6K,L). These results suggest that a loss of CK2α in the hematopoietic lineage may alter the level of antigen-presenting cells in mouse lymphoid tissues.

### 3.6. CSNK2A1 Expression in Human and Mouse Hematopoietic Cells

We evaluated CSNK2A1 expression in human and mouse hematopoietic cells at different maturation stages based on curated microarray data using a published database, Bloodspot 3.0 [61]. In humans, *CSNK2A1* is expressed differentially throughout multiple hematopoietic lineages, including hematopoietic stem and progenitor cells (HSPCs) Figure S3A,C). HSCs and megakaryocyte/erythroid progenitor (MEP) cells showed a higher expression of *CSNK2A1* compared to other cell stages (Figure S3B). In mice, we saw similar patterns with high *CSNK2A1* expression seen in Pro-erythrocyte progenitorsFigure S3B,C). These results show that there is genus-specific (mouse and human) differential expression of CSNK2A1 in hematopoietic lineage cells (Figure S3C).

### 3.7. Deficiency of CK2α Alters Multiple Genes Responsible for HSC Function

**Figure 7:**
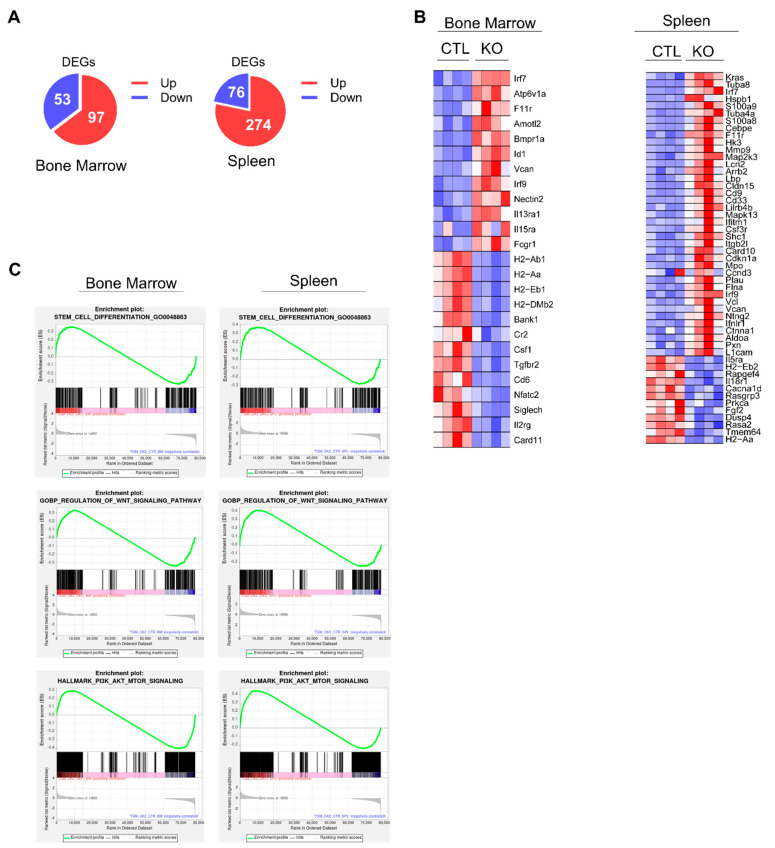
Analysis of gene expression changes in CK2α KO mice. (**A**) Pie chart showing the number of DEGs in CK2α KO BM and spleen cells compared to WT mice cells. (**B**) Heat map showing significantly upregulated and downregulated (*p* < 0.05; fold change ≥ 1.2 and less than or equal to −1.2) genes in CK2α-deficient BM and spleen cells compared to WT mice. (**C**) Gene set enrichment analysis plots of stem cell differentiation genes, Wnt signaling pathway, and PI3K-AKT-mTOR pathway between CK2α KO and WT BM and spleen cells.

We performed RNA sequencing (RNA-seq) followed by differential gene expression analyses of BM and spleen cells from Vav-Cre CK2α^f/f^ and control mice to investigate the potential molecular mechanisms by which CK2α regulates hematopoiesis. The results showed a set of 150 differentially expressed genes (DEGs) in CK2α-deficient BM cells, of which 97 genes were upregulated, and 53 genes were downregulated. Similarly, a loss of CK2α in the spleen samples exhibited a set of 350 DEGs with 274 upregulated and 76 downregulated genes (Figure 7A) (*p*  <  0.05; fold change ≥ 1.5 and less than or equal to −1.5). The loss of CK2α in the BM and spleen samples induced the dysregulation (downregulation) of multiple HSC-maintaining genes, including H2-Ab1, H2-Aa, H2-Eb1, and H2-DMB2 (Figure 7B). These genes belong to pathways involved in HSC function (Figures S4 and S5). Furthermore, gene set enrichment analysis showed significant stem cell differentiation and gene set enrichment along with stem cell maintenance and differentiation signaling pathways, including Wnt, PI3K, AKT, and MTOR in CK2α-deficient BM and spleen cells (Figure 7C). These results suggest that CK2α deficiency alters the molecular signature of pathways involved in HSC differentiation and maintenance.

## 4. Discussion

Previous studies demonstrated the involvement of CK2α as a pro-survival kinase in malignant B-acute lymphoblastic leukemia (ALL) and lymphomas [62,63]. CK2α is overexpressed and essential for cell growth in several types of leukemia and lymphoma [15,33,64]. CK2 is hyperactive and involved in PI3K/PTEN/AKT signaling cascade activation in leukemic conditions [65]. CK2 positively regulates STAT3- and NF-κB-dependent signaling in mantle cell lymphoma [34]. The global deletion of CK2α is embryonically lethal since CK2 is involved in embryogenesis. Wei et al. showed the importance of CK2a in the regulation of B-cell development and differentiation by depleting B-cell specific CK2α [24]. The B-cell specific deletion of the β regulatory subunit of CK2 in mice led to defects in B cell activation pathways [40]. Further, Larson et al. showed that a selective depletion of CK2α expression in myeloid cell populations did not alter their development [41]. We have generated novel conditional knockout mice lacking CK2α in the hematopoietic compartment and described their phenotype. 

We discovered that the deletion of CK2α in the hematopoietic compartment did not alter the mouse phenotype. However, the CK2α cKO mice exhibited paler bones and an enlarged spleen with an increased total cell number. CK2α cKO mice had significantly lower total red blood cell counts and platelets. We found that the increased spleen size correlated with increased erythropoiesis in the spleen, suggesting that the absence of CK2α may exert stress on the spleen to undergo extramedullary hematopoiesis. Our finding is in line with a previous study, which showed that the B-cell specific deletion of CK2α mice exhibited an increase in spleen size [24]. Moreover, it has been well established that several stress conditions can trigger stress-induced erythropoiesis in the spleen, and it serves as a niche for HSCs [66,67]. This was further substantiated by histopathological analyses. H&E staining of BM and spleen sections from CK2α KO mice showed increased myelopoiesis and an altered M:E ratio compared to control mice. Further, we discovered that BM and spleen samples from CK2α-deficient mice exhibited altered HSCs (LSK) and subpopulations, including LT-HSCs, ST-HSCs, and MPPs. 

BM is the main site of hematopoiesis in humans after birth and throughout adulthood [68,69]. During hematopoietic demand or BM failure, substantial hematopoiesis occurs in a number of tissues, including the spleen in a process termed extramedullary hematopoiesis [70,71]. Similarly, BM and the spleen represent remarkable enrichment sites for hematopoiesis in mice [59,72]. Progenitors, including CMPs, GMPs, and MEPs, are derived from LK cells, and CLPs are derived from LSK population expressing IL-7R alpha [59]. Our findings from BM and spleens from mice deficient in CK2a have altered progenitors, indicating that CK2α is involved in the regulation of HSCs and HSCPs. It is well established that both hematopoietic and immune cells originate from a common hematopoietic or lymphopoietic stem cell [73,74]. HSCPs have the ability to differentiate into different immune cells of both myeloid and lymphoid origin [75]. Interestingly, the lack of CK2α in the hematopoietic compartment exhibited decreased T cells and an increased B cell population in the spleen without altering T and B cell populations in the BM. On the other hand, the absence of CK2α skewed towards the myeloid population, including pre- and mature myeloid populations and DCs. Previous reports of the B cell-specific deletion of CK2α in mice exhibited an abnormal distribution within the splenic microenvironment with increased marginal zone B cells and decreased TrB cells [24]. Another study also including B cell-specific CK2β deletion presented with an increased marginal zone and a reduction in follicular B cells [40]. These reports suggest that CK2α has an important but non-essential role in the development and differentiation of B cells. It is well known that CK2 activity regulates a number of signaling pathways involved in stem cell differentiation and immune cell development, including the PI3K/AKT/mTOR, Wnt/b-catenin, NF-κB, and JAK/STAT pathways [1,3,76]. Although CK2 is not an oncogene, it supports oncogenic potential by activating key signaling pathways that prompt the expansion of mutated cancer cells [33,62]. However, taken alone, its expression and activity levels are not sufficient to explain a malignant phenotype. Moreover, unlike HSCs, cancer cells become more dependent on CK2, which makes them more sensitive to a decrease in CK2 activity [32]. Hence, CK2 blockades using a specific inhibitor unraveled its involvement in the JAK/STAT, NF-κB, and AKT pathways in leukemic stem cells [32]. Furthermore, our data support CK2α mediation of the stem cell differentiation pathway, the Wnt signaling pathway, and the PI3K/AKT/mTOR signaling pathway. A growing body of evidence supports CK2 inhibition as an effective strategy to target drug-resistant stem cells in various leukemias [36,37,38,39]. The availability of clinical-grade small-molecule inhibitors of CK2 creates a promising clinical development path for the clinical translation of these findings. The availability of reagents such as this animal model will accelerate investigations to study the role of CK2α in hematopoiesis, in physiologic conditions, and when challenged with oncogenic stimulation. 

## 5. Conclusions

Our data suggest that *CSNK2A1* is dispensable for adult mouse hematopoiesis. However, there are significant changes in the HSC cell compartment that indicate the role of CK2α in HSC functions such as cell cycle regulation and differentiation. Functional studies, such as bone marrow transplantation assays, limited dilution assay and proliferation assays, can decipher CK2α’s regulation of HSC function in baseline and stress conditions [77]. 

## Data Availability

The RNA seq data are deposited in publicly available database. The raw data supporting the conclusions of this article will be made available by the authors on request. The original contributions presented in this study are included in the article/supplementary material. Further inquiries can be directed to the corresponding author.

## Supplementary Materials

The following supporting information can be downloaded at: https://www.mdpi.com/article/10.3390/cells14130963/s1, Table S1: Primers used for the genotype of mice; Table S2: Antibodies used for Western blotting; Figure S1: Gating strategy for immunophenotype analysis of hematopoietic stem cells. (A) CK2 substrate phosphorylation, pAkt s129 and Phospho NFκB p65 western blot from representative mice from CK2α wild type, CK2α cKO and CK2α het mice.1-4 WT, 5-8 cKO, 9-11 Het (B) BM or spleen cells from WT or KO mice were stained with fluorochrome labelled antibodies specific for different cell surface markers and live cells were gated for various hematopoietic lineages. (C) Respective Fluorescence Minus One (FMO) controls were used for final FACS analysis; Figure S2: Gating strategy for immunophenotype analysis of immune cells. (A), BM or spleen cells from WT or KO mice were stained with fluorochrome-labelled antibodies specific for different cell surface markers and live cells were gated for various immune cell types. (B), Respective FMO controls were used for final FACS analysis; Figure S3: CSNK2A1 expression in human and mouse hematopoietic cells. (A) Expression plot of CSNK2A1 expression in hierarchical tree of hematopoietic cells at different maturation stages based on curated microarray data in human and (B) mouse. (C) jitter strip chart of CSNK2A1 expression in human hematopoietic cells (up) and mouse hematopoietic cells (down). These data were generated using the publicly accessible Bloodspot database. BloodSpot 3.0: a database of gene and protein expression data in normal and malignant hematopoiesis Nucl. Acids Res. (2024) doi: 10.1093/nar/gkad993; Figure S4: Differentially expressed genes in bone marrow of CK2α cKO and wild type mice belong to several pathways involved in HSC function. (A) upregulated, (B) downregulated; Figure S5: Differentially expressed genes in spleen of CK2α cKO and wild type mice belong to several pathways involved in HSC function. (A) upregulated, (B) downregulated.
