# Supplementary material for: CK2α Deletion in the Hematopoietic Compartment Shows a Mild Alteration in Terminally Differentiated Cells and the Expansion of Stem Cells
Source: Cells. 2025 Jun 24;14(13):963. doi: 10.3390/cells14130963 (PMC12248783; doi:10.3390/cells14130963)
Supplement: Supplementary file 1 [file cells-14-00963-s001.zip › cells-3661440-supplementary.pdf]

CK2α deletion in the hematopoietic compartment shows a mild alteration in terminally differentiated cells and expansion of stem cells

Rajesh Rajaiah[1], Muhammad Daniyal[1], Marudhu Pandiyan Shanmugam[1], Hannah Valensi[1], Koby Duke[1a], Katie Mercer[1b], Morgann Klink[1c], Lanza Matthew[2], Yasin Uzun[1], Suming Huang[1], Sinisa Dovat[1] and Chandrika Gowda Behura[1*]

[1]Department of Pediatrics, Milton S Hershey Medical Center, Hershey, PA 17033.

[2]Department of Comparative Medicine, Pennsylvania State University College of Medicine Hershey, PA 17033.

*Corresponding author

Dr. Chandrika Gowda Behura

Email: cxg40@psu.edu

Tel: 717-531-6012

[a]Present address: Eurofins Lancaster Laboratories, Lancaster, PA 17601.

[b]Present address: Maynard Children's Hospital at ECU Health Medical Center

[c]Present address: AstraZaneca, Gaithersburg, MD 20878

Table S1: Primers used for the genotype of mice.

	Forward primer	Reverse primer	Probe
Csnk2a1-FL	ACATATTGACGCCTTTTTATACATGTCTCTT	TTCGTATAATGTATGCTATACGAAGTT	TTCAGAGGATATAATGGATAGGCT
Csnk2a1-WT	CAATGTCAAGAGTTACTTGGAATGTAGA	CTCTTTGACCACATCCTAACTATCCCTT	ACGCCTTTTTATACATGTCTCTT
iCre	TCCTGGGCATTGCCTACAAC	CTTCACTCTGATTCTGGCAATTTCG	ACCCTGCTGCGCATTG

Table S2: Antibodies used for Western blotting.

Target	Clone	Number	Manufacturer
CK2 α	E-7	SC-373894	Santa Cruz
CK2 α’	D-7	SC-514403	Santa Cruz
CK2 β		Ab76025	Ab Cam
CK2 sub		87385	Cell signaling
pAKT (s129)	D4P7F	13461s	Cell signaling
Phospho-NF-κB	93H1	3033s	Cell signaling

Table S3: Antibodies used for Flow cytometry

Panels	Targets	Number	Manufacturer
Live/dead	Aqua zombie (BV 510)	423101	Bio legend
Erythroid panel	Anti CD45 (BV 711)	103147	Bio legend
	Anti Ter119 (FITC)	116206	Bio legend
	Anti CD71 (PE)	113808	Bio legend
HSC panel	Anti CD135 (BV 421)	135315	Bio legend
	Anti Lin1 (FITC)	78022	Bio legend
	Anti c-Kit (APC)	105812	Bio legend
	Anti CD127/IL-7Ra (APC-Cy7)	135040	Bio legend

	Anti CD34 (PE-Cy5)	119312	Bio legend
	Anti Sca1 (PE)	122508	Bio legend
	Anti CD16/32 (PE CY7)	101318	Bio legend
Immunophenotyping Panel	Anti CD45 (PE-Cy5)	103110	Bio legend
	Anti CD3 (APC)		Bio legend
	Anti CD19 (BV421)	115549	Bio legend
	Anti NKp46 (PE-Texax red)	137630	Bio legend
	Anti CD11b (BV605)	101257	Bio legend
	Anti F4/80 (BV711)	123147	Bio legend
	Anti CD11c (APC-Cy7)	117324	Bio legend
	Anti MHC-II (Alexa Flour)	107622	Bio legend
	Anti Ly6G (PE-Cy7)	127618	Bio legend
	Anti Ly6C (FITC)	128022	Bio legend

Abbreviations:

CK2 Casein Kinase II

LSC leukemia stem cells

Vav-iCreCK2$^{f/f}$ Hematopoietic specific CK2α-deficient mice

HSC Hematopoietic stem cells

HPSC Human pluripotent stem cells

GEMM Genetically engineered mouse model

KO Knockout

Het	Heterozygous
WT	Wild type
BM	Bone marrow
SDS-PAGE	Sodium dodecyl sulfate-polyacrylamide gel electrophoresis
TBST	Tris buffered saline with Tween 20
PVDF	Polyvinylidene fluoride
HRP	Horseradish peroxidase
RBC	Red blood cell
PBS	Phosphate buffered saline
FBS	Fetal bovine serum
FACS	Fluorescence activated cell sorting
H&E	Hematoxyline and Eosin
FPKM	Fragments Per Kilobase of transcript per Million mapped reads
TMM	Trimmed mean M-values
BH	Benjamini-Hochberg
GSEA	Gene set enrichment analysis
cKO	CK2 knockout
M:E ratio	Myeloid to erythroid

ProE	Proerythroblasts
EryA	Erythroblast A
EryB	Erythroblast B
EryC	Erythroblast C
LSK	Lin^{-}Sca1^{+}c-Kit^{+}
LK	Lin^{-}c-Kit
CMPs	Common myeloid progenitors
GMPs	Granulocyte macrophage progenitors
MEPs	Megakaryocyte erythroid progenitors
LT-HSCs	Long term HSCs
ST-HSCs	Short term HSCs
MPPs	Multipotent progenitors
CLP	Common lymphoid progenitors
DCs	Dendritic cells
DEGs	Differentially expressed genes
ALL	Acute lymphoblastic leukemia

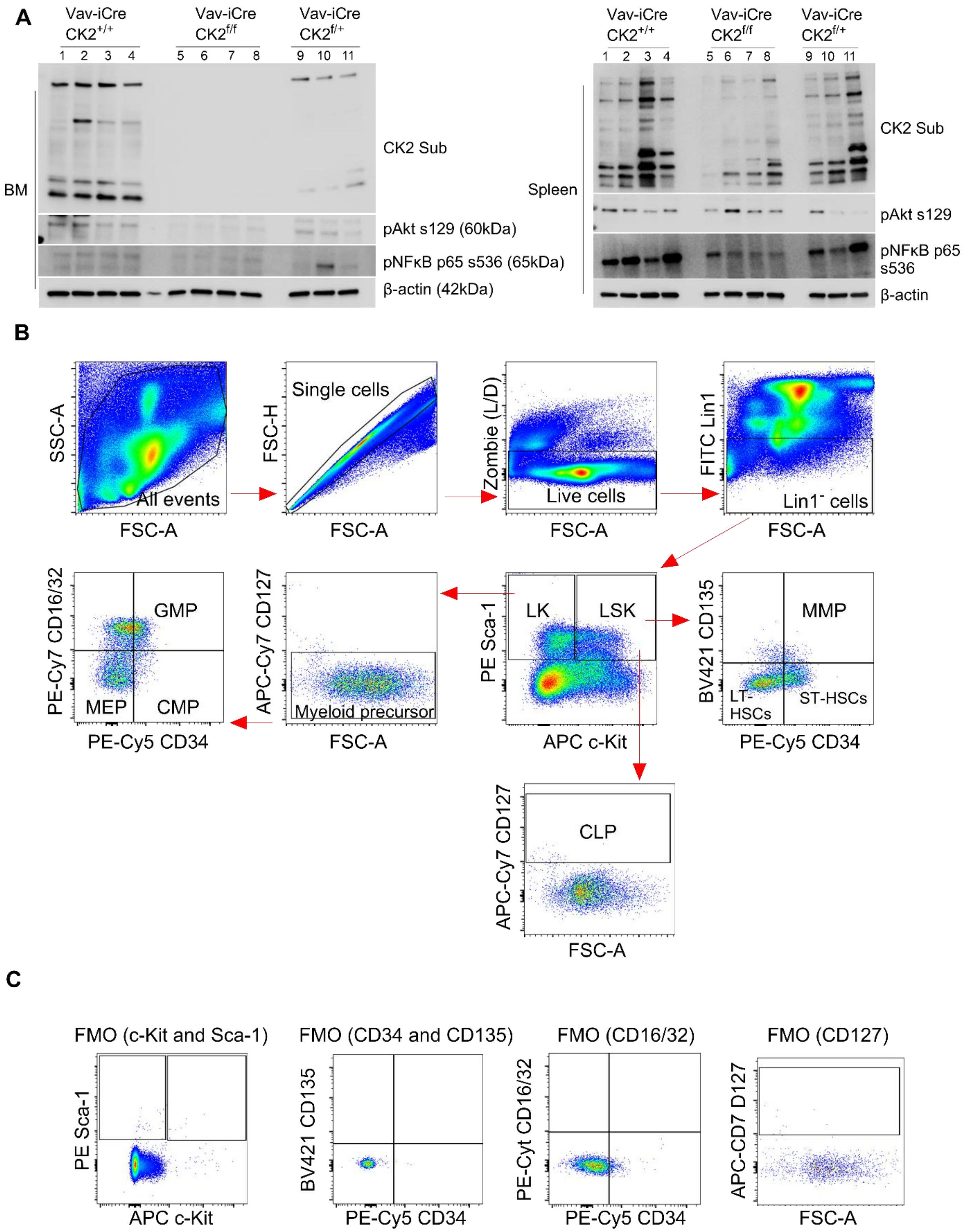
A
Vav-iCre CK2$^{+/+}$
Vav-iCre CK2$^{f/f}$
Vav-iCre CK2$^{f/+}$
1 2 3 4 5 6 7 8 9 10 11
BM
CK2 Sub
pAkt s129 (60kDa)
pNFκB p65 s536 (65kDa)
β-actin (42kDa)
Spleen
CK2 Sub
pAkt s129
pNFκB p65 s536
β-actin
B
SSC-A
FSC-A
All events
FSC-H
Single cells
Zombie (L/D)
Live cells
FITC Lin1
Lin1^{-} cells
PE-Cy7 CD16/32
GMP
MEP
CMP
PE-Cy5 CD34
APC-Cy7 CD127
Myeloid precursor
PE Sca-1
LK
LSK
APC c-Kit
BV421 CD135
MMP
LT-HSCs
ST-HSCs
CLP
C
FMO (c-Kit and Sca-1)
FMO (CD34 and CD135)
FMO (CD16/32)
FMO (CD127)
PE Sca-1
APC c-Kit
BV421 CD135
PE-Cy5 CD34
PE-Cyt CD16/32
APC-CD7 D127
FSC-A

Supplemental Figure S1. Gating strategy for immunophenotype analysis of hematopoietic stem cells. **(A)** CK2 substrate phosphorylation, pAkt s129 and Phospho NFκB p65 western blot from representative mice from CK2α wild type, CK2α cKO and CK2α het mice.1-4 WT, 5-8 cKO, 9-11 Het **(B)** BM or spleen cells from WT or KO mice were stained with fluorochrome labelled antibodies specific for different cell surface markers and live cells were gated for various hematopoietic lineages. **(C)** Respective Fluorescence Minus One (FMO) controls were used for final FACS analysis.

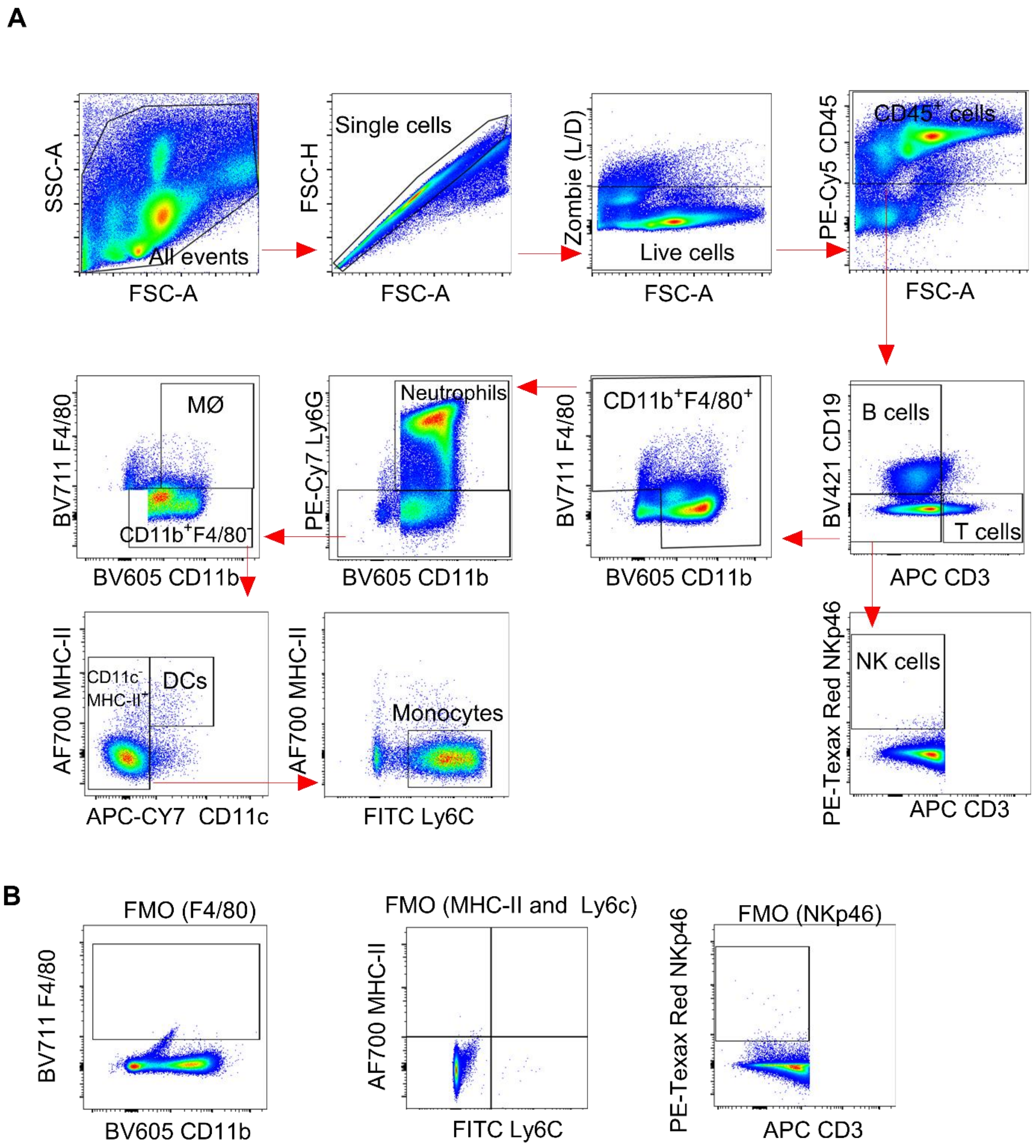

Supplemental Figure S2. Gating strategy for immunophenotype analysis of immune cells. **(A)**, BM or spleen cells from WT or KO mice were stained with fluorochrome-labelled antibodies specific for different cell surface markers and live cells were gated for various immune cell types. **(B)**, Respective FMO controls were used for final FACS analysis.

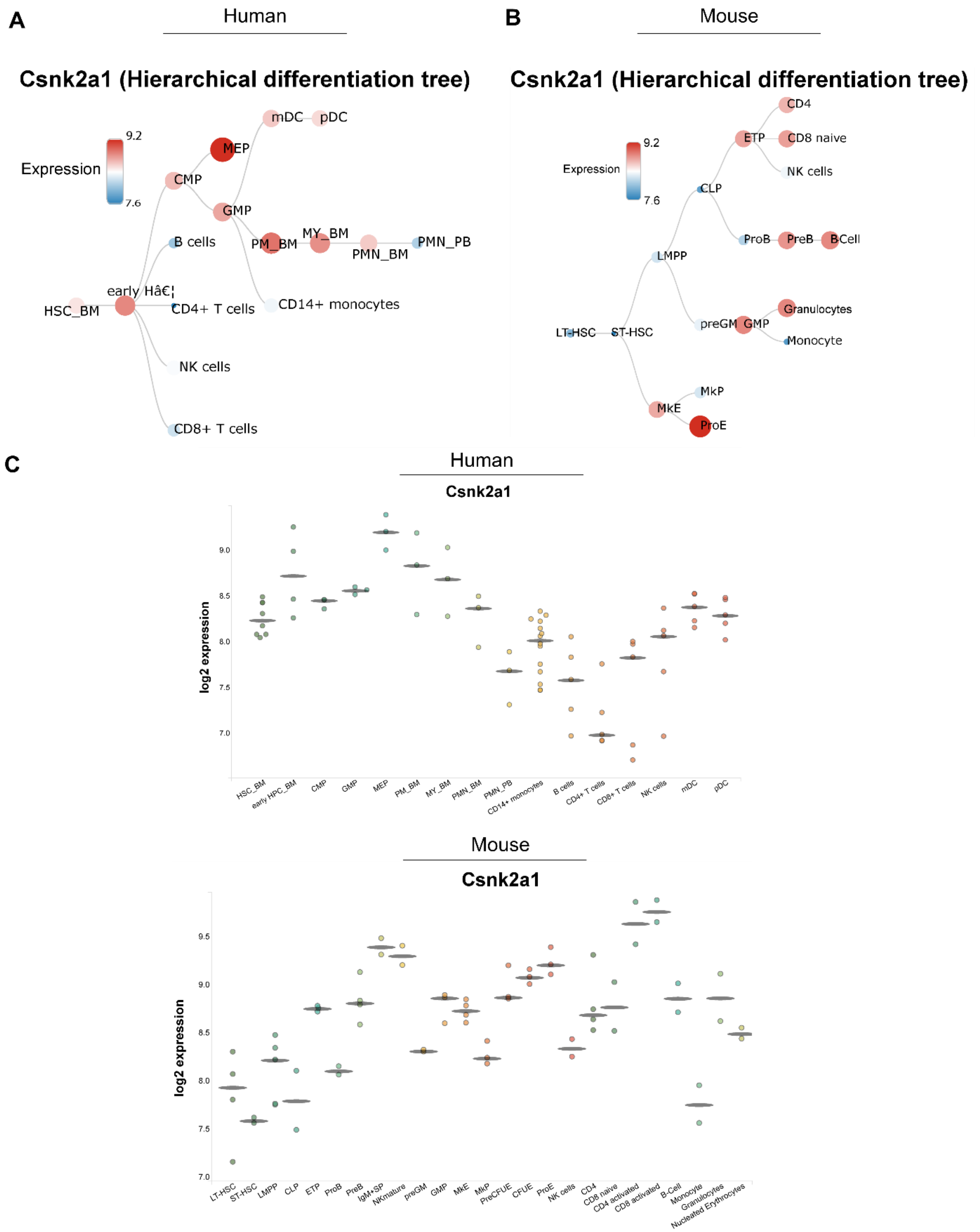

A
Human
Csnk2a1 (Hierarchical differentiation tree)
Expression
9.2
7.6
mDC
pDC
MEP
CMP
GMP
B cells
PM_BM
MY_BM
PMN_BM
PMN_PB
early Hâ€¦
HSC_BM
CD4+ T cells
CD14+ monocytes
NK cells
CD8+ T cells
B
Mouse
Csnk2a1 (Hierarchical differentiation tree)
Expression
9.2
7.6
CD4
ETP
CD8 naive
NK cells
CLP
ProB
PreB
BCell
LMPP
Granulocytes
preGM
GMP
Monocyte
LT-HSC
ST-HSC
MkP
MkE
ProE
C
Human
Csnk2a1
log2 expression
9.0
8.5
8.0
7.5
7.0
HSC_BM
early HPC_BM
CMP
GMP
MEP
PM_BM
MY_BM
PMN_BM
PMN_PB
CD14+ monocytes
B cells
CD4+ T cells
CD8+ T cells
NK cells
mDC
pDC
Mouse
Csnk2a1
log2 expression
9.5
9.0
8.5
8.0
7.5
7.0
LT-HSC
ST-HSC
LMPP
CLP
ETP
ProB
PreB
IgM+SP
NKmature
preGM
GMP
MkE
MkP
PreCFUE
CFUE
ProE
NK cells
CD4
CD8 naive
CD4 activated
CD8 activated
B-Cell
Monocyte
Granulocytes
Nucleated Erythrocytes

Supplemental Figure S3. *CSNK2A1* expression in human and mouse hematopoietic cells. (A) Expression plot of CSNK2A1 expression in hierarchical tree of hematopoietic cells at different maturation stages based on curated microarray data in human and **(B)** mouse. **(C)** jitter strip chart of CSNK2A1 expression in human hematopoietic cells (up) and mouse hematopoietic cells (down). These data were generated using the publicly accessible Bloodspot database. BloodSpot 3.0: a database of gene and protein expression data in normal and malignant hematopoiesis Nucl. Acids Res. (2024) doi: 10.1093/nar/gkad993

A (Bone marrow upregulated genes)

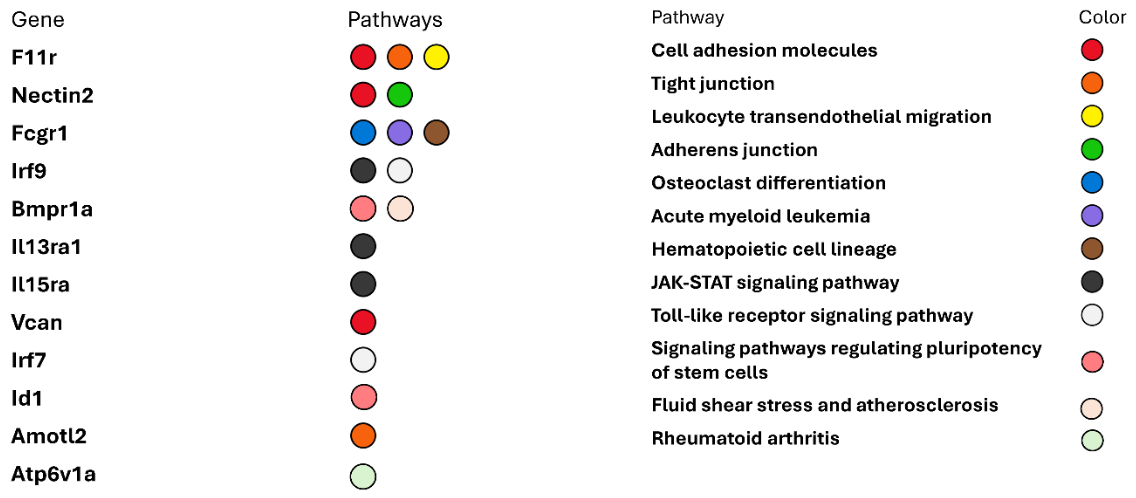

B (Bone marrow downregulated genes)

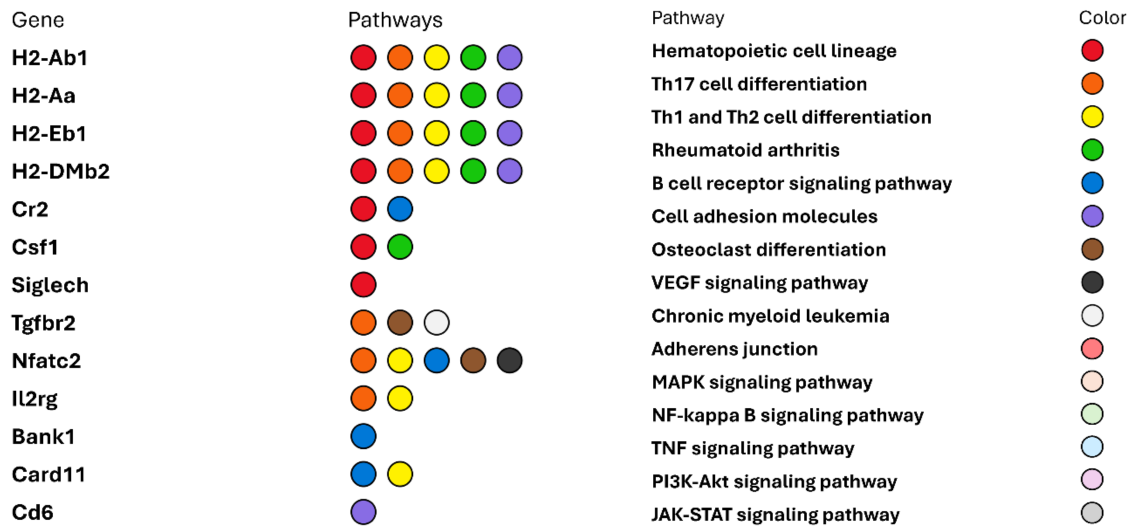

Supplemental Figure S4. Differentially expressed genes in bone marrow of CK2α cKO and wild type mice belong to several pathways involved in HSC function. (A) upregulated, (B) downregulated.

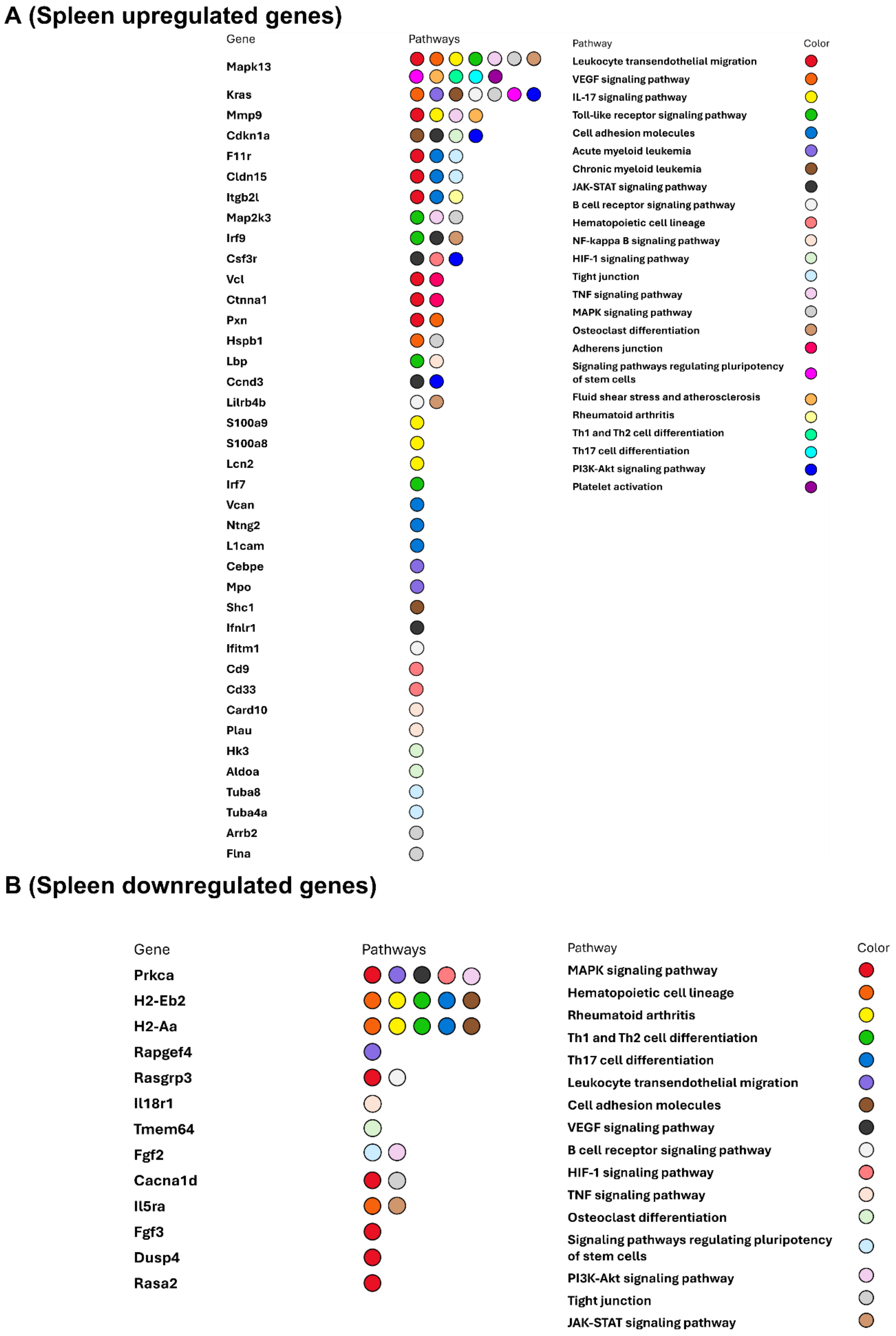
A (Spleen upregulated genes)
Gene
Pathways
Mapk13
Kras
Mmp9
Cdkn1a
F11r
Cldn15
Itgb2l
Map2k3
Irf9
Csf3r
Vcl
Ctnna1
Pxn
Hspb1
Lbp
Ccnd3
Lilrb4b
S100a9
S100a8
Lcn2
Irf7
Vcan
Ntng2
L1cam
Cebpe
Mpo
Shc1
Ifnlr1
Ifitm1
Cd9
Cd33
Card10
Plau
Hk3
Aldoa
Tuba8
Tuba4a
Arrb2
Flna
Pathway
Color
Leukocyte transendothelial migration
VEGF signaling pathway
IL-17 signaling pathway
Toll-like receptor signaling pathway
Cell adhesion molecules
Acute myeloid leukemia
Chronic myeloid leukemia
JAK-STAT signaling pathway
B cell receptor signaling pathway
Hematopoietic cell lineage
NF-kappa B signaling pathway
HIF-1 signaling pathway
Tight junction
TNF signaling pathway
MAPK signaling pathway
Osteoclast differentiation
Adherens junction
Signaling pathways regulating pluripotency of stem cells
Fluid shear stress and atherosclerosis
Rheumatoid arthritis
Th1 and Th2 cell differentiation
Th17 cell differentiation
PI3K-Akt signaling pathway
Platelet activation
B (Spleen downregulated genes)
Gene
Pathways
Prkca
H2-Eb2
H2-Aa
Rapgef4
Rasgrp3
Il18r1
Tmem64
Fgf2
Cacna1d
Il5ra
Fgf3
Dusp4
Rasa2
Pathway
Color
MAPK signaling pathway
Hematopoietic cell lineage
Rheumatoid arthritis
Th1 and Th2 cell differentiation
Th17 cell differentiation
Leukocyte transendothelial migration
Cell adhesion molecules
VEGF signaling pathway
B cell receptor signaling pathway
HIF-1 signaling pathway
TNF signaling pathway
Osteoclast differentiation
Signaling pathways regulating pluripotency of stem cells
PI3K-Akt signaling pathway
Tight junction
JAK-STAT signaling pathway

Supplemental Figure S5. Differentially expressed genes in spleen of CK2α cKO and wild type mice belong to several pathways involved in HSC function. (A) upregulated, (B) downregulated.
